# “We need good nutrition but we have no money to buy food”: sociocultural context, care experiences, and newborn health in two UNHCR-supported camps in South Sudan

**DOI:** 10.1186/s12914-018-0181-3

**Published:** 2018-11-12

**Authors:** Stephanie Gee, Josep Vargas, Angel M. Foster

**Affiliations:** 1 United Nations High Commissioner for Refugees, Case Postale 2500, CH-1211 Genève 2, Dépôt Switzerland; 2 University of Ottawa, 1 Stewart Street, 312-B, Ottawa, ON K1N6N5 Canada

**Keywords:** Maternal and newborn health, Refugees, Sudan, South Sudan, Traditional practices, Qualitative research

## Abstract

**Background:**

Determinants of newborn health and survival exist across the reproductive life cycle, with many sociocultural and contextual factors influencing outcomes beyond the availability of, and access to, quality health services. In order to better understand key needs and opportunities to improve newborn health in refugee camp settings, we conducted a multi-methods qualitative study of the status of maternal and newborn health in refugee camps in Upper Nile state, South Sudan.

**Methods:**

In 2016, we conducted 18 key informant interviews with health service managers and front-line providers and 13 focus group discussions in two Sudanese refugee camps in Maban County, South Sudan. Our focus group discussions comprised 147 refugee participants including groups of mothers, fathers, grandmothers, traditional birth attendants, community health workers, and midwives. We analysed our data for content and themes using inductive and deductive techniques.

**Results:**

We found both positive practices and barriers to newborn health in the camps throughout the reproductive lifecycle. Environmental and contextual factors such as poor nutrition, lack of livelihood opportunities, and insecurity presented barriers to both general health and self-care during pregnancy. We found that the receipt of material incentives is one of the leading drivers of utilization of antenatal care and facility-based childbirth services. Barriers to facility-based childbirth included poor transportation specifically during the night; insecurity; being accustomed to home delivery; and fears of an unfamiliar birth environment, caesarean section, and encountering male health care providers during childbirth. Use of potentially harmful traditional practices with the newborn are commonplace including mixed feeding, use of herbal infusions to treat newborn illnesses, and the application of ash and oil to the newborn’s umbilicus.

**Conclusions:**

Numerous sociocultural and contextual factors impact newborn health in this setting. Improving nutritional support during pregnancy, strengthening community-based transportation for women in labour, allowing a birth companion to be present during delivery, addressing harmful home-based newborn care practices such as mixed feeding and application of foreign substances to the umbilicus, and optimizing the networks of community health workers and traditional birth attendants are potential ways to improve newborn health outcomes.

## Background

Neonatal mortality is a key indicator of overall population health and has broad social and behavioural determinants, reaching across the reproductive life cycle. Defined as any death that occurs in the first 28 days of life, neonatal mortality accounts for over 45% of all deaths of children under 5 years globally [1]. Many of these deaths occur during the first day or week of life, and most are preventable with known interventions [2]. In addition to availability and accessibility of quality health services, broader determinants of health impact newborn outcomes including gender inequality, low maternal education levels, poverty, and malnutrition [3]. Humanitarian emergencies and poor baseline socioeconomic conditions often coexist, making displaced women and newborns particularly vulnerable due to disruptions in health services; limited access to livelihoods; risks of sexual violence; and language and cultural barriers. Despite this, previous reviews have shown that reproductive health indicators such as maternal and neonatal mortality are often better in refugee camp settings than in the host population, likely due to a concentration of health services and other resources within the camps [4, 5]. Nonetheless, many barriers persist and target indicators are often not met, including for services that impact newborn health such as antenatal and postnatal care utilization, skilled birth attendance, caesarean section rates, and early and exclusive breastfeeding [4, 6]. Understanding the factors that influence maternal and newborn care decisions is crucial for improving health programming in acute and protracted refugee settings.

In January 2016, the United Nations High Commissioner for Refugees (UNHCR) launched the “Saving Newborn Lives in Refugee Settings” project in refugee camps in Kenya, South Sudan, and Jordan. Supported by the Bill and Melinda Gates Foundation, the project aimed to gain a better understanding of the local contexts in order to identify priorities for intervention related to newborn health. The overarching assessment included a scoping review of the anthropological literature, health facility assessments in a selection of camps, and a qualitative exploration of the knowledge, beliefs, and practices of Somali, Sudanese, and Syrian refugees as they relate to newborn health. In this paper, we focus on the qualitative findings related to sociocultural context and care experiences from two Sudanese refugee camps in South Sudan.

### South Sudan

South Sudan is both a source country of refugees as well as a host to refugees fleeing violence in neighbouring countries. As a result of the escalation of violence in both Sudan and South Sudan, at the end of 2015 there were more than 263,000 refugees and almost 1.7 million internally displaced persons in South Sudan [7]. Sudanese refugees began fleeing across the border to Upper Nile State in South Sudan in 2011 to escape fighting between the Sudanese Armed Forces and the Sudanese People’s Liberation Movement-North (SPLM-N) in South Kordofan and Blue Nile States. As a result, around 129,559 Sudanese refugees from Blue Nile state have settled in four refugee camps in Upper Nile State [7], including the Yusuf Batil and Doro camps, where our baseline assessment took place. The Ingassana and Urduk tribes predominate Yusuf Batil and Doro camp populations; these communities are characterized by small-scale agriculture (around 70%), nomadic pastoralism, and, for the Ingassana, artisanal gold mining [8, 9].

South Sudan has some of the world’s worst national maternal and newborn health indicators: the maternal mortality ratio is 789 per 100,000 live births, the neonatal mortality rate is 39 per 1000 live births, only 19% of births are attended by a skilled birth attendant [10], and the caesarean section rate is less than 1% [11]. In the two targeted camps, neonatal mortality rates for the year prior to the study (2015) ranged from 5.9 per 1000 in Doro camp to 50 per 1000 in Yusuf Batil. This difference was due in part to lack of 24/7 skilled delivery in Yusuf Batil camp during part of the year due to security concerns, resulting in 40% of women in this camp delivering without a skilled birth attendant [12]. Challenges in providing aid in the camps are many, including on-going insecurity, the multiplicity of armed groups, attacks on both civilians and humanitarian workers, tensions between host and refugee populations, high staff turnover, shortage of qualified health manpower, and logistical issues due to poor roads and infrastructure [13].

#### Reproductive beliefs and practices

Very little published research focuses on Blue Nile populations. Literature on reproductive beliefs and practices in Sudan and South Sudan indicate that childbearing is highly valued within this region, with women’s status and identities strongly intertwined with maternity [14, 15]. Early marriage [16] and large families are desirable due to high child mortality and in order to help with household chores, assist parents in old age, and confer high social status on the family [17]. Family and tribal ties are forged through the husband’s payment of a bride-price (often in the form of cattle) with the expected return on investment being many children [18]. Use of contraceptives is uncommon as not bearing children results in strong social disapproval [19]. Pregnancy and childbirth are viewed as natural processes and thus women may forgo routine antenatal care, even if services are available [20]. There is a tendency toward fatalism, that is, ascribing both the inability to obtain health care and adverse pregnancy outcomes to the will of God [21, 22]. Traditionally, births take place at home with a traditional birth attendant (*daya* in South Sudan) who provides physical and emotional support [16, 23, 24].

#### Postnatal care practices

Research from rural Sudan indicates that women often remain in the home for 40 days after delivery, during which time they may forgo postnatal care and follow-up visits for the neonate [20]. Although breastfeeding is religiously and culturally encouraged and is an accepted method of birth spacing [16, 20], *exclusive* breastfeeding rates are low in both South Sudan and Sudan at 45 and 55%, respectively [10, 25]. In Upper Nile state and other parts of South Sudan, sexual taboos in the postpartum period may facilitate birth spacing; couples achieve the expected 2–3 year period of sexual abstinence after the birth of a child by living separately or the man taking another wife. This abstinence period corresponds with the period of breastfeeding and relates to a traditional belief that sex during the breastfeeding period will bring a curse upon the child or family [16].

#### Care-seeking behaviours

Women often seek care from multiple systems of healing, including traditional birth attendants (TBAs), village surgeons (*arets*), home remedies, and biomedical care [20, 26, 27]. Traditional practitioners may be sought due to personal beliefs or because biomedical health services are not available. However, little is known about traditional medicine beliefs and practices among the numerous ethnic groups [15]. Factors such as rural location, illiteracy, low income, and poor quality, availability, and access to health services contribute to low rates of preventative and curative care in the region, including routine antenatal and postnatal care [15].

## Methods

We conducted both semi-structured in-depth interviews with key informants and focus group discussions in order to explore a range of issues related to maternal and newborn health. In order to focus on participants’ lived experiences, beliefs, and practices we employed a phenomenological approach [28].

### Data collection

Data collection occurred in February 2016 in Doro and Yusuf Batil camps in Maban County, South Sudan. SG supervised data collection and conducted most key informant interviews. We trained teams of facilitators to conduct the FGDs; each team comprised one moderator and two note takers. Facilitators were local South Sudanese student nurses who were able to speak local languages and English. Professional midwives were trained to assist us with the health facility assessments; they also conducted some of the key informant interviews.

### Sampling

We used both purposive and convenience sampling for the key informant interviews. We identified key informants in leadership positions through publicly available information. We also invited a sub-set of front-line health workers who were working at the health facility on the day of the health facility assessment to participate in an interview. No one declined to participate.

For the FGDs, NGO partners in the camps used word-of-mouth to recruit participants. As we wanted to understand the perspectives of a range of community members, we purposively recruited mothers with children under the age of two, fathers with children under the age of two, grandmothers, TBAs, community health workers, and midwives. We formed our FGDs around these relational and professional characteristics.

### Key informant interviews

We conducted in-depth interviews with 18 key informants (Table 1) in English, with the help of a translator when necessary. After receiving written informed consent, we conducted interviews in private offices within the health facilities. The interview guide explored perspectives on health priorities, experiences providing care, and perceptions of strengths, challenges, and needs related to health service provision. Based on participant preference we either audio-recorded or took extensive notes during the interviews. We also recorded numerous informal discussions and observations in a field journal throughout the assessment process.

**Table 1:** Number and type of key informants

Type of participant	Number of interviews
Health program manager	5
Front-line health worker	8
United Nations public health official	5
Total	18

### Focus group discussions

We held 13 FGDs in Doro and Yusuf Batil refugee camps, with a total of 147 participants (Table 2). This included four groups with mothers, two groups with fathers, grandmothers, and community health workers, and one group (in Yusuf Batil camp) with midwives. Each group consisted of 8–12 participants and lasted an average of 1.5 h. Prior to the FGDs, the facilitators, who were members of the local community, adapted questions and agreed upon culturally appropriate language and expressions. We conducted FGDs in the local language, with note takers simultaneously recording notes in English. Following each session, the facilitators and SG compiled notes, reviewed the data, and agreed on key findings.

**Table 2:** Composition and location of focus group discussions

	Number of FGDs in Doro Camp	Number of FGDs in Yusuf Batil Camp	Number of participants
Mothers	2	2	44
Fathers	1	1	22
Grandmothers	1	1	22
TBAs	1	1	24
CHWs	1	1	27
Midwives	–	1	8
Total	6	7	147

### Data analysis

Data analysis involved an iterative process that coincided with data collection. SG reviewed all notes, transcribed audio-recordings, and read these materials repeatedly before generating an initial codebook. This allowed us to gain a holistic understanding of the data and increase familiarity. We used both inductive and deductive approaches to conduct content and thematic analyses [29, 30]. We derived codes from the data and systematically coded content line-by-line. After completing this initial coding, we combined codes into broader organizing themes and the organized these themes by general area of inquiry, which were pre-determined (Table 3). In the final analytic phase we compared the results of the two components of the study to identify concordant and discordant findings. We used NVIVO 10 to manage our data during the coding process and help organize themes.

**Table 3:** Codes, themes, and areas of inquiry

Area of inquiry	Organizing theme	Codes
Preconception	Family dynamics and the refugee experience	Preferred family size Drivers of family size Restrictions of refugee camp life Birth spacing Family decision making Suspicion over injectable contraceptive
Antenatal	Nutrition and livelihoods	Limited livelihoods Cuts in food aid Cuts in supplementary feeding program for pregnant women
Childbirth	Material incentives and facility delivery	Incentives for facility delivery Birth notification Refusal of those ‘born on the way’
	Barriers to facility delivery	Donkey-cart ambulance Security fears Fear of referral (unfamiliar environment, caesareans, male provider) Ease and familiarity with home delivery
	Shifting role of traditional birth attendant	New role of TBAs TBA support in antenatal TBA support in childbirth and postpartum Attitudes of TBAs
Postnatal	Newborn feeding practices	Traditional practices Colostrum Mixed feeding Changes since arriving in camp
	Umbilical and thermal care	Lalobe seed ash/charcoal/oil for umbilicus Thermal care practices Kangaroo mother care
	Care seeking for the newborn	Clinic care Traditional remedies for the newborn

In the results section, we organize our findings around stages in the reproductive cycle (preconception, antenatal, childbirth, and postnatal) as these reflect our areas of inquiry. We then present the most salient themes and provide representative quotes to illustrate our findings. We have redacted and masked all personally identifying information.

## Results

### Preconception period

#### Family dynamics and the refugee experience

Our FGDs participants reported preferences for a large number of children (often 10 or more) in order to help with household chores, increase family income, and gain status within the community. However, restrictions of refugee camp life appear to be impacting desired family size. Housing, food, and livelihoods are severely restricted in the camps, and refugees are dependent upon humanitarian aid to meet their basic needs. Violent conflicts between refugees and the host population over resource use, such as collecting firewood, have placed further strains on the population. As one participant in the FGD with mothers explained, “In the north [Blue Nile State] there was no problem – you could have as many as you wanted, but here in Doro it is difficult to have many children. We lack income – they cannot all eat well.”

Groups discussed the issues of birth spacing and use of contraceptives openly. Although most participants stated that the number of children was “God’s will” and contraceptive prevalence rates are low, family planning did not seem to be a stigmatizing subject. Both men and women extolled the benefits of family planning, including increasing the time to breastfeed a child, allowing the child to grow and be healthy, allowing the woman time to rest, and creating less financial burden for the family. Many participants referenced a two-year space between babies as optimal and birth spacing methods used included the tradition of postpartum abstinence, breastfeeding, and the “injectable.” As one participant in the FGD with fathers in Yusuf Batil notes, “Some women are tired from delivery. If so, she can use the injectable.” In our discussions with both mothers and grandmothers, some women raised perceptions of contraceptive coercion by health service professionals. As one mother in Doro camp explained, “They never ask us about family planning, but sometimes they give it to us without permission, when we don’t want it. If they see your children are too close in age they take you and inject you without your permission.”

Both FGD participants and key informants reported that decisions around family planning and other family decisions are generally under the authority of the husband, although many noted the importance the husband and wife making joint decisions. However, men rarely, if ever, attend postnatal visits where family planning is discussed.

### Antenatal period

#### Nutrition and livelihoods

Participants in all discussion groups reported that nutrition was both the predominant “general health concern” as well as the main method of “self care” during pregnancy. Lack of food was a significant stressor for FGD participants and was raised frequently in the groups. At the time of our study the population relied heavily on food aid, however allocated food rations had been cut by 30% due to funding constraints. Camp residents had few options for self-sufficiency, such as collecting of firewood for sale, leaving much of the camp population hungry. Community members recognized the need for pregnant women to eat nutritious foods, avoid heavy work, and reduce household chores but women struggled to do so. As a pregnant participant in an FGD in Doro camp explained:

*We need good nutrition but we have no money to buy food. You can force yourself to go into the woods to get firewood to get some to sell, but you may fall, you are carrying heavy things, and you may also get arrested and your axe taken away for cutting wood.*

Cuts in funding also resulted in the cancellation of the Blanket Supplementary Feeding (BSF) program for pregnant and lactating women. One of the mothers in Doro Camp lamented the diminished services:

*These days they have stopped everything. They just take the MUAC [mid-upper arm circumference] and then say: ‘You are ok, you are not malnourished, just go home.’ Why don’t they provide the food in time, before there is a problem?*

### Childbirth period

#### Material incentives and facility use

Given the dire poverty in the camps, uptake of preventative care and facility-based childbirth services appears to be closely linked to the receipt of material incentives for attendance. Mothers acknowledged the importance of attending antenatal care (ANC) visits and highly valued the incentives they received, including mosquito nets and BP5 (high energy) biscuits; the amount and type of incentive varied depending on the provider. Staff at the local Ministry of Health hospital, who were not currently able to provide incentives due to funding issues, reported decreasing attendance:

*In 2012–2013 we were having a high number of antenatals. But nowadays we have no soap, no mosquito nets, no mamma kits, so the number of ANC visits are reducing. They are now going to the others. The NGOs are having all these things but we don’t.*

Postnatal care visits, which are not incentivized, notably saw lower attendance than antenatal care visits.

Similarly, the most frequently mentioned reason for choosing facility delivery over home delivery was due to the receipt of the birth notification certificate. Getting this directly from the hospital facilitates the child’s registration with UN agencies and eases acquisition of a ration card. Secondly, receiving “mamma kits” containing basic supplies like mosquito netting, soap, a basin, and cloth were highly sought after. Participants in our FGDs also acknowledged that the skill of doctors and midwives in providing safe care also motivated women to deliver in facilities.

Despite the successes, the incentivizing of facility delivery has added to tensions between health workers and parents. Mothers and fathers in our FGDs expressed feeling discriminated against for delivering “on the way” to the facility or at home, regardless of the reason for the delivery site or their intentions. Mothers and fathers also reported being turned away and scorned by health facility staff when arriving at the facility with their freshly birthed newborn, and not receiving immediate postnatal care. As one mother in a Doro camp FGD explained, “Many of them [pregnant women] try to go by foot but give birth on the way. If you deliver at home there is no help. They [health facility staff] will say, ‘It’s up to you. It’s not our problem, because you delivered at home.’” A father, also in Doro camp, stated, “If your wife has contractions, take her to the health centre. But if she delivers on the way they won’t accept her.”

A number of the health workers that we spoke with, both key informants and FGD participants, reported that they believe some parents are purposefully coming late in order to access some of the material incentives offered to promote facility births without actually delivering in-facility. Regardless of the validity of this belief, this dynamic appears to be restricting access to immediate postnatal care for the mother and newborn, as well as creating a reciprocal mistrust between patients and providers.

#### Barriers to facility delivery

The main barriers to facility delivery related to unreliable transportation, security concerns, comfort with home delivery, and fears of an unfamiliar birthing environment. The camp-based emergency transport system for women in labour (a donkey-cart) was not working well in either camp resulting in delays or refusals of transportation. Women in labour are advised to first contact the traditional birth attendant (TBA), who then assists her to arrange a donkey cart, which is often kept at the home of the sheikh or umdah (clan leader). Security fears due to recent violent attacks in Yusuf Batil camp also made residents hesitant to leave home at when in labour at night. As one participant in an FGD with mothers explained, “It was night and I couldn’t get to the hospital. My husband went to the sheikh to call for the donkey cart, but he refused, so I delivered at home, with a traditional birth attendant. My plan was to deliver at the facility.”

Women and health workers also reported a fear of upward referral from the primary health centre in the camp to the secondary facility outside of the camp, related to lack of familiarity with the facility, fear of caesarean section, and fear of being treated by a male health worker.

#### The shifting role of the traditional birth attendant

The role of the traditional birth attendant is gradually shifting in the community. NGO health service providers discourage home deliveries and encouraged TBAs to act as “companions”, assisting the labouring woman to reach the health facility. However, the role of “companion” does not permit the TBA to remain with the woman once she is admitted to the health facility.

The TBAs who participated in our FGDs described their traditional role as a supportive one, with care extending from the antenatal period through delivery and into the postnatal period. During pregnancy they may provide porridge and extra food to the woman or advocate to her family and husband to do so. TBAs report that this has become increasingly difficult due to limited food availability in the camps. When the woman is in labour, TBAs provide porridge, hot water for the woman to bathe, help position the woman, and assist with the delivery. They described performing episiotomies with a razor blade if the baby’s head is big, and tying the umbilical cord with a piece of grass or thread from a sack. They use scissors or a piece of sharp grass to cut the cord, depending what they have on hand, and then apply charcoal from the Lalobe tree mixed with sesame oil, or charcoal from a burned coir sack, to the umbilical stump. They clear away any mucous from the baby’s nose and mouth and put the baby skin-to- skin with mother. If the baby is not breathing they describe holding the baby upside down and hitting him on the back or feet. If the woman is bleeding after delivery they “push on the abdomen to make the blood come out”.

TBAs in the FGDs stated that they support facility delivery and are happy in their new roles. However, they feel unfairly blamed by health care providers if a woman decides to deliver at home and later requests their help. They would like to be able to remain with the mother during the delivery in the facility and are also eager to learn more skills from the midwives.

### Postpartum period

#### Infant feeding practices

Both key informants and FGD participants described early, but not exclusive, breastfeeding as the traditional newborn feeding practice in this community; women typically supplement breast milk with animal milk, water, and porridge. Commercial infant formula and bottles are generally not used or available. Prior to arriving in the camps (within the last 4 years), the norm was to discard the colostrum. As one TBA in Yusuf Batil camp explained, “In Blue Nile [source location] we wouldn’t breastfeed the baby until it reaches one week of age, and give water and porridge until then.”

Mothers and grandmothers described how their understanding of infant feeding practices have changed considerably since arrival in the camps as a result of health promotion messages. Colostrum is increasingly recognized to be healthy and both grandmothers and mothers in FGDs stated that they now wait until 6 months before introducing solid foods.

#### Umbilical cord and thermal care

The most common method of newborn umbilical cord care is burning the seed of the Lalobe tree then grinding it, mixing the resultant charcoal with sesame oil, and applying the mixture to the umbilicus. Health workers appear to give little advice to families on how to care for the umbilicus at home.

Mothers and grandmothers expressed a good understanding and appreciation for thermal care practices in the newborn period and described keeping the baby well wrapped, using warm water to wash the baby, and keeping the newborn close to the mother at all times. Although extended skin-to-skin (Kangaroo Mother Care or KMC) for premature babies was initially unfamiliar to community members, word-of-mouth and observation of the practice in the postnatal ward in Doro camp has increased its acceptability among community members. As one mother described, “If the baby is born in the seventh month, put the baby between your breasts so it can get warm. [The NGO] will give you blankets to cover you.”

There were no specific cultural barriers raised to the practice of KMC by community members. Potential barriers raised by health workers include fatigue or illness of the mother, lack of understanding of the method, and lack of private space in facilities.

#### Care-seeking and traditional medicines

Women generally follow the traditional practice of 40 days of rest in the home following delivery, during which time women are able to recuperate from the birth, bond with the baby, and receive help with newborn care from elder female family members. Community members also described a tradition of a three-month home confinement for premature babies. Women do not perceive the preventative care offered as part of routine postnatal care (PNC) as a priority. However, curative health services are well regarded in the camps and participants noted the kindness of doctors and their good skills and knowledge. Long waiting times due to overcrowding and dissatisfaction with the medications were the main deterrents to clinic care. While the health clinic was the first choice of care provider for a sick newborn, grandmothers continue to prefer the care of traditional healers. Both key informant and FGD participants report that traditional medicines are commonly used in routine home care of the newborn. In addition to using the charred Lalobe seed for umbilical care, a variety of local seeds and roots are used for newborn illnesses such as diarrhoea (Nabak tree roots or Kirua bush roots pounded and mixed with water to form a paste for ingestion), malaria (Ceremarium tree seeds), and fever (topical application of Kamun seeds in oil). A cloth bracelet filled with Kamun (cumin) seeds may also be tied around the baby’s wrist to be sucked upon. Another traditional practice described includes feeding soil from an ancestor’s grave to the baby to prevent diarrhoea. Similarly, when moving to a new place, parents feed the baby a small amount of dirt from the ground of the new location to prevent diarrhoea.

## Discussion

Understanding local sociocultural and contextual factors is crucial for identifying potential barriers and facilitators to newborn health and survival at the home and community level, and developing targeted and locally relevant interventions to improve outcomes. In Maban camps, despite the difficult conditions, community members generally hold positive perceptions of health services and are receptive to health promotion messages, including for facility-based childbirth and improved infant feeding practices. At the same time, numerous challenges to exist related to insecurity, limited options for livelihoods and self-sufficiency, poor nutrition, shortage of food due to cuts in food rations, and certain harmful home-based newborn care practices. As well, despite their availability, key services such as ANC, facility delivery, and PNC continue to be underutilised [12].

### Removing barriers to facility-based delivery

Skilled birth attendance is a key factor in improving both maternal and neonatal outcomes [2]. Our findings support previous service reviews in refugee camps which found notable shifts in cultural norms towards facility delivery [6]. In Maban camps, although refugee women stated that they preferred health facilities for delivery, a cohort of women continue to deliver at home. Similar to many other low-income settings, the “ease” of home delivery coupled with transportation difficulties are leading reasons why women opt for home deliveries [31]. In Maban camps we found the donkey-cart ambulance for women in labour, although a community asset, also created barriers for women trying to reach a health facility as it was frequently unavailable to them. Working with key community leaders to improve access to the donkey-cart ambulances, as well as developing a birth and emergency plan with the expectant mother during antenatal care visits are important steps to address this barrier. Insecurity in the camps also made expectant parents hesitate to leave home during labour, particularly at night. In such cases, the provision of clean delivery kits for use in the home, containing soap, sterile blade and cord ties, clean plastic sheet, and gloves, with clear instructions on their use, would reduce some potential risks of home delivery when it is unavoidable.

Fears of an unfamiliar birth environment, caesarean section, and being treated by male staff were raised as reasons why women may reject facility delivery, or may not agree to be transferred to a higher level of care. These findings are very similar to those expressed by refugee women in our reviews of Somali refugee camps in Kenya [32]. Further, these results are in line with a qualitative evidence synthesis of 34 studies which found that unfamiliar or undesirable birth practices, lack of privacy, lack of social support, and fear of cutting (episiotomy or caesarean section) were common factors that decreased facility-based delivery in low and middle-income countries [31]. Allowing a birth companion of the women’s choice to accompany her through labour and delivery is a simple method to increase women’s comfort, improve experiences of care, and improve newborn outcomes [33, 34]. Given that TBAs who are supportive of health facility delivery are available in Maban, allowing them to remain with the labouring woman in the health facility to provide emotional support and comfort may be a simple and low-cost step to help to improve facility delivery rates and provide more culturally appropriate services.

### The use of incentives to increase utilization of maternal services

The simple material incentives such as soap and mosquito nets that are used to encourage attendance at ANC visits and the birth notification and “mamma kits” received for facility delivery appear to be effective in increasing utilization. A number of other studies have shown the effectiveness of non-monetary material incentives to increase the uptake of health services [35–37]. However, incentives are of limited use if other key barriers, such as transport and security, are not addressed. Further, the inconsistency of incentives provided by different providers (for example, NGOs versus the Ministry of Health) results in disparities between camp residents, and between refugee and host populations. Similarly, providing incentives for only select services (for example, ANC but not PNC) may inadvertently send a message about their relative importance. In this setting, incentives have also led to reciprocal mistrust between health providers and patients; some health workers fear that they are being manipulated and in some cases are refusing to provide care. Given these complexities, it is important that incentives be standardized across care providers in close proximity to one another in order to avoid creating or deepening inequities. It is also critical that incentives not be used in a punitive way, a dynamic that fuels distrust between patient and health provider.

### Impacting home-based maternal and newborn care practices

Maternal nutrition, breastfeeding, and thermal and umbilical care are key contributors to newborn health and survival [2]. Poor nutrition emerged as a general health concern for FGD participants, as well as the leading concern during pregnancy. Notably, at the time of our study, global acute malnutrition rates were up to 15% in South Sudan camps (critical) and the anaemia rates for women of reproductive age and children 6–59 months were up to 55% (high) and 31% (medium-high), respectively [38]. The withdrawal of nutrition programs for pregnant and lactating women was particularly frustrating to women and families. There is a pressing need to ensure reliable and balanced nutritional support for pregnant women as well as increased opportunities for livelihoods in the camps.

While breastfeeding is a primary method of infant feeding, early and exclusive breastfeeding is not the traditional norm. However, participants described a shift away from harmful traditional practices such as discarding colostrum, delayed initiation of breastfeeding, and mixed feeding of the newborn, following health promotion efforts in the camps. Umbilical cord care practices continue to be strongly dominated by potentially harmful traditional methods and specifically the application of ash from the Lalobe tree or charcoal and oil. Community members and health workers alike seem generally unaware of recommended umbilical care practices, indicating a need for increased education on this subject. Similar to other studies in the region [20, 26, 27], traditional remedies are often used concurrently with biomedical health seeking and are particularly popular among grandmothers who may choose them ahead of clinic-based services, suggesting a need for increased health promotion efforts that include the extended family.

Studies have shown that community-based interventions, including health promotion groups and home visits by trained community health workers, can be effective in reducing stillbirths and neonatal mortality, increasing referrals for care in the case of pregnancy complications, and improving breastfeeding practices [39]. The camps in Maban benefit from a network of community health workers who are themselves refugees and therefore familiar with local languages, beliefs, and customs. Increasing their skills and knowledge in maternal and newborn health and implementing an organized system of home visits in the antenatal and postnatal periods may be valuable to improve infant feeding and umbilical care practices. This approach also allows the involvement of elder females and husbands in health promotion messages, which is important given the strong influence that elder female social networks have on important practices such as pregnancy care, place of delivery, family nutrition, and infant feeding practices [40].

### Limitations

This study has a number of limitations. We completed the overarching assessment, including the qualitative pieces, in only two refugee camps in South Sudan; we did not explore the experiences of urban or non-camp refugees. This necessarily limits the transferability of our findings. Although we conducted FGDs in the local language, simultaneous note taking in English may have led to translation errors and a loss of depth of meaning. Time constraints resulted in shorter than desired training sessions for focus group facilitators. Further, the limited timeframe for our fieldwork meant that we could not use thematic saturation as an end point for data collection. We reviewed our preliminary findings with field-level staff but we did not review our findings and recommendations with community-based participants; future research might benefit from more robust member checking. It is recognized that the roles of the first two authors, being both UNHCR staff and conducting data collection, may have influenced the responses of participants. Finally, the positionalities of our team members, including gender, nationality, race, profession, and educational level, undoubtedly influenced our reporting and interpretation. Daily supervision, regular debriefings among team members, reflexive memoing in the field journal, and triangulation helped us minimize these limitations and increase the credibility and trustworthiness of our findings.

## Conclusion

In order to improve newborn health outcomes, in addition to ensuring quality health services, service providers must have an understanding of the sociocultural and contextual environment that shape beliefs and practices and impact health service utilization. This study has identified numerous factors across the reproductive lifecycle that, if effectively addressed, have the potential to improve outcomes. Improving nutritional support and community-based emergency transportation, allowing a birth companion to be present during delivery, addressing harmful home-based newborn care practices such as mixed feeding and the application of foreign substances to the umbilicus, and optimizing the skills and knowledge of community health workers to conduct home visits are important areas of focus moving forward. Past successes have promoted healthier infant feeding practices and improved facility-based delivery rates thus indicating the community’s receptiveness to health promotion messages and the potential for continued improvements.

## Abbreviations

ANC: Antenatal care
BSF: Blanket Supplementary Feeding
FGD: Focus group discussion
KMC: Kangaroo Mother Care
MUAC: Mid-upper arm circumference
NGO: Non-governmental organization
PNC: Postnatal care
SPLM-N: Sudanese People’s Liberation Movement-North
TBA: Traditional birth attendant
UNHCR: United Nations High Commissioner for Refugees
